# Knowledge, attitude and prevalence of ivermectin use as coronavirus disease treatment: A crosssectional study among a Malaysian population

**DOI:** 10.51866/oa.415

**Published:** 2024-01-06

**Authors:** Tar'ali Filza Nur Athirah, Abdul Jalil Aina Amanina, Nor Afendi Nor Safwan Hadi

**Affiliations:** 1 BPharm, MClin Pharm, Faculty of Pharmacy and Health Sciences, Royal College of Medicine Perak, Universiti Kuala Lumpur, Ipoh, Perak, Malaysia. Email: aina.amanina@unikl.edu.my; 2 BPharm, Faculty of Pharmacy and Health Sciences, Royal College of Medicine Perak, Universiti Kuala Lumpur, Ipoh, Perak, Malaysia.; 3 BPharm, MClin Pharm, Faculty of Pharmacy and Health Sciences, Royal College of Medicine Perak, Universiti Kuala Lumpur, Ipoh, Perak, Malaysia.

**Keywords:** Knowledge, Attitude, Prevalence, Ivermectin, COVID-19

## Abstract

**Introduction:**

During the coronavirus disease (COVID-19) pandemic, there was a widespread public misconception regarding ivermectin use in managing the disease. There is no approval of ivermectin as COVID-19 treatment by the Food and Drug Administration, and the adverse effects of the drug are alarming. This study aimed to evaluate the knowledge and attitude towards and prevalence of ivermectin use as COVID-19 treatment among the Malaysian population.

**Methods:**

This cross-sectional study was conducted among 391 Malaysians aged ≥18 years. A validated online self-administered questionnaire disseminated via Google Forms was used to evaluate the knowledge and attitude towards and prevalence of ivermectin use as COVID-19 treatment. The socio-demographic characteristics and knowledge and attitude towards and prevalence of ivermectin use among the respondents were evaluated through descriptive analysis. The chi-square test was used to identify the association between the variables.

**Results:**

The respondents had moderate levels of knowledge and attitude towards ivermectin use, while the prevalence of ivermectin use was 3.58%. The respondents’ sex (P=0.014), age (P=0.012) and monthly income (P=0.049) were significantly associated with their level of knowledge. The respondents’ sex (P=0.04) was significantly associated with their level of attitude. Conversely, no socio-demographic characteristics were significantly associated with the prevalence of ivermectin use as COVID-19 treatment.

**Conclusion:**

The majority of Malaysians have moderate levels of knowledge and attitude towards ivermectin use as COVID-19 treatment, with a low prevalence of actual use.

## Introduction

In 2020, severe acute respiratory syndrome coronavirus 2, one of the many types of coronaviruses, caused coronavirus disease (COVID-19), which was declared a pandemic respiratory illness by the World Health Organization.^1^ Several repurposed antiviral drugs, including remdesivir, hydroxychloroquine, lopinavir and interferon beta-1a, have been tested as potential treatments for COVID-19. However, hydroxychloroquine and lopinavir have not been found to reduce the mortality rate of patients with COVID-19 and have been therefore dropped from clinical trials. In contrast, remdesivir has shown promising results in reducing the recovery time of patients with COVID-19 in some clinical trials. Taken together, current evidence suggests that repurposed antiviral drugs may have moderate effects on the outcomes of patients with COVID-19, but further research is needed to establish their effectiveness.^2^

A previous retrospective study involving 5889 clinical records of all reverse-transcription polymerase chain reaction-confirmed COVID-19 cases among patients aged ≥12 years in Malaysia reported a prevalence of mild disease of up to 92% with a low fatality rate of 1.2%. Hydroxychloroquine was administered to over 37% of patients, mainly those with severe illness. Approximately 77% of patients with severe illness received antiviral treatment, particularly lopinavir/ritonavir. Steroids and tocilizumab were used sparingly, predominantly for more severe cases.^3^

During the initial phase of the COVID-19 pandemic, there was a shortage of effective treatments and vaccines. This led to a search for potential therapies, with some studies suggesting that ivermectin could be a viable option. However, there has been controversy surrounding the use of ivermectin for COVID-19 owing to conflicting evidence and concerns about its effectiveness. While ivermectin has shown antiviral properties in laboratory settings, its efficacy in treating COVID-19 remains uncertain. Other drugs such as azithromycin, chloroquine and hydroxychloroquine have also faced similar controversies and have been de-implemented by health authorities owing to concerns about their effectiveness. Despite these recommendations, the use of these drugs has persisted partly owing to misinformation on social media and challenges in enforcing guidelines. Overall, while ivermectin has potential, there is insufficient evidence to support its use as COVID-19 treatment.^4^

On 22 December 2021, the Food and Drug Administration issued an emergency use authorisation for Pfizer’s nirmatrelvir/ritonavir (Paxlovid) to be used as COVID-19 treatment in adult and paediatric patients with mild-to-moderate infection. Nirmatrelvir/ritonavir is one of the COVID-19 treatments in the form of a pill to be taken orally and is designated to be administered twice daily for 5 days.^5^ The cost per 5 days of treatment is USD 250, which is approximately RM 1092.50. The expensive cost as well as the non-availability in the market has led to a preference for ivermectin as COVID-19 treatment over nirmatrelvir/ritonavir.

The outbreak of COVID-19 has brought global recognition to ivermectin. This recognition is attributed to its possible mechanism of action, which involves its ability to suppress viruses and host nuclear import of proteins.^6^ However, in a randomised clinical trial performed in Malaysia among 490 patients, the administration of ivermectin during the early phase of infection was not found to prevent the disease progression to severe COVID-19. Accordingly, the authors did not recommend the use of ivermectin for patients with COVID-19.^7^

According to the Ministry of Health (MOH) Malaysia,^8^ ivermectin is registered in the country as an antiparasitic drug that can be administered only to animals to treat parasitic infections such as onchocerciasis, strongyloidiasis and helminthiases. No ivermectin product has been approved for human use in Malaysia. There is evidence of sales and public consumption of ivermectin in the country, contrary to the warning by the MOH on the adverse effects of the drug. Individuals who strongly believe in its prophylactic efficacy against COVID-19 may go to great lengths to acquire this medication, even resorting to unconventional means, given that it is classified as a prescription-only medication. Owing to some people’s reluctance to consult physicians and pharmacists, two cases of ivermectin poisoning have been recorded in Malaysia.^9^

Given the abovementioned context, this study aimed to evaluate the knowledge and attitude towards and prevalence of ivermectin use among Malaysians. The findings are expected to provide correct information to the government to foster and increase awareness among Malaysians on the use of ivermectin. In particular, the findings could serve as a groundwork for the government to identify the pattern and practice of ivermectin use among the public to provide a specific target in raising awareness.

## Methods

### Study Design

A cross-sectional study was conducted using a voluntary and anonymous self-administered online structured questionnaire. The questionnaire was disseminated via Google Forms. Respondents’ identities were not divulged during reporting or presentations. The findings of this study, together with any gathered information, were kept strictly confidential, and only abridged data were presented.

### Study population

Non-probability convenience and snowball sampling methods were employed in this study. Adults who were aged ≥18 years, resided in Malaysia during the period of data collection and were willing to participate were included in this study. Adults who could not comprehend the English or Malay language and those who provided an incomplete answer to the questionnaire were excluded.

### Sample size calculation

The Raosoft® sample size calculator^10^ was used to calculate the recommended sample size for this study. According to the Department of Statistics Malaysia, the population size of the country is approximately 32.67 million.^11^ Based on the Raosoft® calculation, the minimum sample size needed in this study was 385 respondents.

### Data collection

The online questionnaire was prepared using Google Forms, and a link address was generated to invite Malaysians to participate in this study. The link was distributed along with a standardised description of the study, which comprised the self-introduction of the researchers, the title of the study, sections of the questionnaire, the aim of the study, informed consent and the contact number of the research team. The link was disseminated through several social media platforms such as WhatsApp, Facebook and Instagram, as these platforms were deemed cost-effective and efficient in achieving the target sample size for this study.

### Research instrument and scoring method

The questionnaire was newly developed and the content was validated before data collection. It consisted of 23 questions divided into four different sections: Section A: sociodemographic characteristics including sex, age, level of education, occupation and monthly income; Section B: prevalence of ivermectin use as COVID-19 treatment; Section C: knowledge regarding ivermectin use; and Section D: attitude towards ivermectin use as prophylaxis therapy for COVID-19.

**Table 1:** CVI among the three experts.

CVI	Expert 1 + 2 + 3
S-CVI/Ave	0.94
S-CVI/UA	0.83
Average proportion of items judged as relevant among the three experts	0.94

CVI, content validity index; S-CVI/Ave, content validity index based on the average method; S-CVI/UA, content validity index based on the universal agreement method

The respondents’ overall levels of knowledge and attitude were categorised using Bloom’s cut-off point: The levels were considered good when the score was 80%–100%; moderate, 60%–79%; and poor, <60%.^12^

### Content validation of the questionnaire

Content validation was conducted to evaluate the relevance, clarity and coherence of the items in the questionnaire based on expert judgements concerning the defined domains of knowledge and prevalence of ivermectin use as COVID-19 treatment. A content validation form was prepared and distributed to three experts in their respective fields from local and public universities in Malaysia. The experts were requested to critically review all domains and their items to determine their relevance before providing a score for each item. The content validity index (CVI) must be at least 0.8 to meet the requirement. Based on the evaluation, the CVI for each question was greater than 0.80, which indicated that it met the criteria (Table 1).

### Pilot study

Prior to data collection, a pilot study was performed among 30 randomly selected Malaysians to identify the feasibility and validity of the questionnaire. The data collected were analysed using Cronbach’s alpha test via the Statistical Package for the Social Sciences (SPSS) version 26 (IBM Corp., Armonk, NY, USA) to test the internal consistency of the research instrument. The Cronbach’s alpha values for Sections C and D were 0.907 and 0.877, respectively.

### Data analysis

The data were recorded and analysed using the SPSS version 26. The socio-demographic characteristics were evaluated through descriptive analysis and were presented as frequencies, percentages and means. The dependent variables, including the knowledge and attitude towards and prevalence of ivermectin use as COVID-19 treatment as well as the source of information regarding ivermectin use as COVID-19 treatment, were also examined using descriptive analysis and presented as frequencies, percentages and means. Next, the chi-square test was used to identify the association of the socio-demographic characteristics with the knowledge and attitude towards and prevalence of ivermectin use. P-values of≤0.05 were considered statistically significant.

## Results

### Socio-demographic characteristics

A total of 391 Malaysians answered the questionnaire that was distributed for 3 months from March to May 2022. The sociodemographic characteristics of the respondents are summarised in Table 2

**Table 2:** Socio-demographic characteristics (N=391).

Characteristics		Frequency	Percentage
Sex	Male	111	28.4
	Female	280	71.6
Age	<18 years	0	0
	18–30 years	311	79.5
	31–60 years	77	19.7
	61–75 years	3	0.8
Level of education	Primary education	1	0.3
	Secondary education	59	15.1
	University or higher	331	84.7
	Others	0	0
Occupation	Student	277	70.8
	Healthcare sector	16	4.1
	Non-healthcare sector	68	17.4
	Unemployed	30	7.7
Monthly income	No income	257	65.7
	Below RM 2500	57	14.6
	RM 2501-5000	36	9.1
	RM 5001-10000	29	7.4
	RM 10001 and above	12	3.1

### Prevalence of ivermectin use as COVID-19 treatment

Among the respondents, 14 (3.6%) consumed ivermectin as COVID-19 treatment, while the remaining (n=377, 96.4%) did not. The mean ± standard deviation (SD) of the prevalence of ivermectin use as COVID-19 treatment was 0.04±0.186, calculated using the following formula:


$$\text{Prevalence}=\frac{\text{Total number of respondents consuming ivermectin as COVID-19 treatment}}{\text{Population size}}\times 100$$

### Knowledge towards ivermectin use as COVID-19 treatment

Tables 3 displays the respondents’ knowledge of ivermectin use as COVID-19 treatment. The majority of the respondents (73.4%) knew that ivermectin is an antiparasitic drug. Most respondents (n=273, 69.8%) knew that ivermectin is used to treat scabies caused by *Sarcoptes scabiei,* onchocerciasis, lymphatic filariasis and other parasitic infections. Only 183 (46.8%) respondents knew that ivermectin is approved for use in animals. Most respondents (n=316, 80.8%) knew that there are insufficient data regarding ivermectin use as COVID-19 treatment. Conversely, approximately half of the respondents (n=221, 56.5%) were aware that ivermectin is not safe to be used as COVID-19 treatment, while 50.1% knew the side effects of ivermectin, including dizziness, vomiting, allergy, seizure, coma and death. The majority of the respondents (n=287, 73.4%) were aware that an overdose of ivermectin can lead to poisoning, and 282 (72.1%) respondents knew that the MOH Malaysia has issued a warning on the consumption of ivermectin as COVID-19 treatment.

**Table 3:** Knowledge of ivermectin use as COVID-19 treatment (N=391).

Knowledge of ivermectin use as COVID-19 treatment	True n (%)	False n (%)	Do not know n (%)	Mean (SD)
1. Ivermectin is under the antiparasitic class of drugs.	287 (73.4)	23 (5.9)	81 (20.7)	0.85 (0.495)
2. Ivermectin is used to treat scabies caused by *Sarcoptes scabiei*, onchocerciasis, lymphatic filariasis and other parasitic infections.	273 (69.8)	17 (4.3)	101 (25.8)	0.79 (0.506)
3. Ivermectin is approved for use in animals.	183 (46.8)	103 (26.3)	105 (26.9)	0.99 (0.730)
4. Ivermectin is approved by the FDA for human use to treat COVID-19.	150 (38.4)	59 (15.1)	182 (46.5)	0.69 (0.720)
5. There are insufficient clinical data regarding ivermectin use as COVID-19 treatment.	316 (80.8)	26 (6.6)	49 (12.5)	0.94 (0.435)
6. Ivermectin is safe, cheap and effective as COVID-19 treatment.	51 (13.0)	221 (56.5)	119 (30.4)	0.83 (0.637)
7. The side effects of ivermectin include dizziness, vomiting, allergy, seizure, coma and death.	196 (50.1)	160 (40.9)	35 (9.0)	0.68 (0.631)
8. An overdose of ivermectin can lead to poisoning.	287 (73.4)	27 (6.9)	77 (19.7)	0.87 (0.500)
9. The Ministry of Health Malaysia has issued a warning on the consumption of ivermectin as COVID-19 treatment.	282(72.1)	24 (6.1)	85 (21.7)	0.84 (0.505)

COVID-19, coronavirus disease; SD, standard deviation; FDA, Food and Drug Administration.

Each response was given a score of 1 for the correct answer option and a score of 0 for the incorrect and do not know answer options. The mean ± SD of the knowledge score of the respondents was 5.61±1.958. Only 52 (13.3%) respondents had good knowledge regarding ivermectin use as COVID-19 treatment. Overall, majority of the respondents had a moderate level of knowledge regarding ivermectin use as COVID-19 treatment (n=172, 44%) based on a knowledge score of 62.33% (5.61/9×100).

### Attitude towards ivermectin use as COVID-19 treatment

A total of 129 (33.0%) respondents were neutral about ivermectin being used in treating COVID-19. Of the respondents, 107 (27.4%) strongly disagreed that ivermectin is safe to be consumed, while 65 (16.6%) agreed. Conversely, 114 (29.2%) respondents strongly disagreed; 122 (31.2%) were neutral; and 14 (3.6%) strongly agreed that ivermectin can protect them against COVID-19. A total of 102 (26.1%) respondents strongly agreed that the sale of ivermectin for human use should be prohibited; conversely, 34 (8.7%) respondents strongly disagreed, while 123 (31.5%) were neutral. Further, 124 (31.7%) respondents strongly disagreed that the MOH should encourage the public to use ivermectin to treat and prevent COVID-19, while 33 (8.4%) agreed. Most respondents (n=236, 60.4%) strongly agreed that it is necessary to obtain information about ivermectin before consumption. Lastly, 233 (59.6%) respondents strongly agreed that they are aware that misuse of ivermectin can lead to serious adverse effects.

The total mean ± SD attitude score towards ivermectin use as COVID-19 treatment was 19.56±4.408. The overall attitude towards ivermectin use as COVID-19 treatment is detailed in Tables 4.

**Table 4:** Attitude towards ivermectin use as COVID-19 treatment (N=391).

Attitude towards ivermectin use as COVID-19 treatment	Strongly agree n (%)	Agree n (%)	Neutral n (%)	Disagree n (%)	Strongly disagree n (%)	Mean (SD)
1. I believe ivermectin should be used in treating COVID-19.	17(4.3)	33 (8.4)	129 (33.0)	116 (29.7)	96 (24.6)	2.62 (1.077)
2. I think ivermectin is safe to be consumed.	14 (3.6)	65 (16.6)	124 (31.7)	81 (20.7)	107 (27.4)	2.52 (1.161)
3. I think ivermectin can protect against COVID-19-	14 (3.6)	56 (14.3)	122 (31.2)	85 (21.7)	114 (29.2)	2.59 (1.153)
4. I believe it is better to prohibit the sale of ivermectin for use in humans.	102 (26.1)	88 (22.5)	123 (31.5)	44 (11.3)	34 (8.7)	2.46 (1.233)
5. The Ministry of Health should encourage the public to use ivermectin to treat and prevent COVID-19.	7 (1.8)	33 (8.4)	135 (34.5)	92 (23.5)	124 (31.7)	2.75 (1.049)
6. It is necessary to obtain information about ivermectin before consumption.	236 (60.4)	73 (18.7)	58 (14.8)	16 (4.1)	8 (2.0)	3.31 (1.002)
7. I am aware that the misuse of ivermectin can lead to serious adverse effects.	233 (59.6)	74 (18.9)	66 (16.9)	12 (3.1)	6 (1.5)	3.32 (0.962)

COVID-19, coronavirus disease; SD, standard deviation

A significant proportion of respondents comprising 43.5% (n=170), indicated a moderate level of attitude toward the use of ivermectin as a COVID-19 treatment. Additionally, 29.2% (n=114) expressed a positive attitude, while 27.4% (n=107) reported a poor attitude.

### Association of the socio-demographic characteristics with the knowledge and attitude towards and prevalence of ivermectin use as COVID-19 treatment

The respondents’ sex (P=0.014) and monthly income (P=0.049) were significantly associated with their knowledge level. In addition, there was a strong association found (P=0.012) between the respondents’ age and knowledge level. Conversely, there was no relationship noted between the respondents’ educational level (P=0.318) and occupation (P=0.166) and knowledge level.

The respondents’ sex (P=0.04) was significantly associated with their attitude level towards ivermectin use. Conversely, the respondents’ age, educational level, occupation and monthly income were not significantly associated with their attitude level.

There was no significant association between the respondents’ sex, age, educational level, occupation and monthly income with the prevalence of ivermectin use as COVID-19 treatment (Table 5).

**Table 5:** Association of the socio-demographic characteristics with the knowledge and attitude towards and prevalence of ivermectin use as COVID-19 treatment.

	Total knowledge score, n (%)					Total attitude score, n (%)				Total prevalence score, n (%)			
	Poor	Moderate	Good	χ^2^ (df)	P	Poor	Moderate	Good	χ^2^(df)	P	Yes	No	χ^2^ (df)	P
Sex				8.471 (2)	0.014				6.439 (2)	0.040			0.383 (1)	0.536
Male	53 (37.1)	52 (30.2)	6(11.5)			39 (36.4)	48 (28.2)	24 (21.1)			5 (35.7)	106 (28.1)		
Female	114 (68.3)	120 (69.8)	46 (88.5)			68 (63.6)	122 (71.8)	90 (78.9)			9 (28.1)	271 (71.9)		
Age				12.764 (4)	0.012				6.288 (4)	0.179			0.397 (2)	0.820
18–30 years	138 (82.6)	129 (75.0)	44 (84.6)			91 (85.0)	126 (74.1)	94 (82.5)			12 (85.7)	299 (179.3)		
31–60 years	29 (17.4)	42 (24.4)	6 (11.5)			16 (15.0)	42 (24.7)	19 (16.7)			2 (14.3)	75 (19.9)		
61–75 years	0 (0.0)	1 (0.6)	2 (3.8)			0 (0.0)	2(1.2)	1 (0.9)			0 (0.0)	3 (0.8)		
Educational level				4.715 (4)	0.318				6.075 (4)	0.194			0.487 (2)	0.784
Primary education	0 (0.0)	1 (0.6)	0 (0.0)			0 (0.0)	1 (0.6)	0 (0.0)			0 (0.0)	1 (0.3)		
Secondary education	22 (13.2)	32 (18.6)	5 (9.6)			14 (13.1)	33 (19.4)	12 (10.5)			3 (21.4)	56 (14.9)		
University or higher	145 (86.8)	139 (80.8)	47 (90.4)			93 (86.6)	136 (80.0)	102 (89.5)			11 (78.6)	320 (84.9)		
Occupation				9.137 (6)	0.166				2.996 (6)	0.809			0.736 (3)	0.865
Student	118 (70.7)	116 (67.4)	43 (82.7)			75 (70.1)	117 (68.8)	85 (74.6)			10 (71.4)	267 (70.8)		
														
Healthcare sector	8 (4.8)	8 (4.7)	0 (0.0)			6 (5.6)	7(4.1)	3 (2.6)			0 (0.0)	16 (4.2)		
Non-healthcare sector	27 (16.2)	37 (21.5)	4 (7.7)			20 (18.7)	31 (18.2)	17(14.9)			3 (21.3)	65 (17.2)		
Unemployed	14 (8.4)	11 (6.4)	5 (9.6)			6 (5.6)	15 (8.8)	9 (7.9)			1 (7.1)	29 (7.7)		
Monthly income				31.474 (20)	0.049				19.210 (20)	0.508			6.825 (10)	0.742
No income	113 (67.7)	111 (64.5)	33 (63.5)			70 (65.4)	112 (65.9)	75 (65.8)			10 (71.4)	247(65.5)		
Below RM 2500	23 (13.8)	19 (11.0)	15 (28.8)			17 (15.9)	20 (11.8)	20 (17.5)			1 (7.1)	56 (14.9)		
RM 2501-5000	19 (11.4)	16 (9.3)	1 (1.9)			9 (8.4)	20 (11.7)	7(6.1)			1 (7.1)	35 (9.2)		
RM 5001-10000	9 (4.4)	17(9.9)	3 (5.7)			8 (7.4)	13 (7.7)	8 (7.1)			1 (7.1)	28 (7.4)		
RM 10001 and above	3(1.8)	9 (5.2)	0(0)			3(1.8)	5 (3.0)	4 (3.5)			1 (1.7)	11 (3.0)		

COVID-19, coronavirus disease

## Discussion

In this study, the respondents’ level of knowledge was categorised as moderate based on a score of 62.33%. The mean ± SD of the knowledge score was 5.61±1.958. In their study conducted in 2021^13^ among 306 healthcare workers, Verma et al. did not classify their respondents’ level of knowledge and presented the results for each knowledge domain question only as frequencies and percentages. The authors asked healthcare workers about whether they knew about COVID-19, whether they knew if there is available treatment for COVID-19, whether they had ever heard about ivermectin, whether they knew about ivermectin’s role in preexposure prophylaxis for COVID-19 and whether they knew that ivermectin use for COVID-19 is still in the experimental phase.

Herein, the mean ± SD of the attitude score towards ivermectin use as COVID-19 treatment was 19.56±4.408. The respondents were categorised to have a moderate attitude level based on a score of 69.85%. The same previous study conducted by Verma et al. in 2021^13^ did not classify whether their respondents had good, moderate or poor attitude levels regarding ivermectin use. The authors only listed their results on each attitude domain question. Approximately 88.24% of healthcare workers agreed that ivermectin should be administered for COVID-19 preexposure prophylaxis; 70% believed that ivermectin can prevent COVID-19; 41.18% indicated that ivermectin can provide them with a false sense of security, as the medicine is still in the experimental stage; 58.82% were aware that ivermectin can produce adverse reactions; and 62.75% indicated that ivermectin use can yield more benefits than risks.

The relationship between public knowledge and attitude towards ivermectin use as COVID-19 treatment is an important aspect to consider when assessing public perceptions and making public health policy decisions. Public knowledge can significantly influence people’s attitudes and behaviours, especially when it comes to healthcare and medicine. Public knowledge about the efficacy and safety of ivermectin in treating COVID-19 may influence their attitudes towards its use. When the general population has a thorough and well-informed understanding from clinical trials, scientific studies and expert opinions related to ivermectin, people may be inclined to avoid its use, as there is no evidence of its efficacy and safety as COVID-19 treatment. Conversely, misinformation, incomplete understanding or lack of awareness can lead to unnecessary ivermectin use.

Conducting surveys to assess public attitude and knowledge about ivermectin can provide valuable information to the MOH and health professionals. The findings of this study could serve as a basis for designing effective awareness campaigns, as the questionnaire used may reveal areas where the public lacks accurate information or has misconceptions about ivermectin. This could help health authorities identify specific issues for education and awareness-raising. When survey results uncover common misconceptions, healthcare professionals can proactively address them through clear and accessible communications. This may include debunking myths and presenting scientific evidence pertaining to the known knowledge regarding ivermectin use.

Owing to the online nature of the survey in this study, there was an over-representation of youth and student respondents (more than 70%), who are more likely to engage in social media than the older population. Early treatment of COVID-19 is generally indicated for people who are aged more than 50 years or have comorbidity owing to their high risk of developing severe disease. Hence, ivermectin use might be more prevalent among older individuals, who were under-represented in this study. Therefore, the present findings might not be generalisable to the entire Malaysian population.

## Conclusion

The adult Malaysians showed moderate levels of knowledge and attitude towards ivermectin use as COVID-19 treatment, with a low prevalence of actual use. These findings are hoped to provide valuable insights that can aid healthcare professionals and relevant authorities in disseminating information regarding the appropriate use of ivermectin, with the ultimate goal of preventing future incidents of ivermectin poisoning. The findings underscore the importance of targeted public health campaigns and education to improve the responsible and safe use of ivermectin. It is evident that a significant proportion of the Malaysian population may benefit from accurate information and guidance on the use of this medication. These insights should inform policymakers, healthcare professionals and relevant stakeholders in developing strategies to ensure the rational use of ivermectin and minimise the risk of misuse and adverse effects in the Malaysian population.

## Data sharing statement

The raw data from this study will be not be shared via controlled access repositories to ensure the protection of participant confidentiality. Interested parties can request access to the data, and upon approval, they will be provided with the necessary information for retrieval. This approach allows for transparency in data sharing while upholding ethical and privacy considerations.

**How does this paper make a difference in general practice?**

- The findings are expected to provide important information to the government and healthcare workers in Malaysia on the need to educate the population on the use of ivermectin.
- The findings could be used to identify sources where the population obtains information about ivermectin use as COVID-19 treatment.
- The findings could also be utilised to determine whether the public gains information on ivermectin use from valid and authentic sources or whether such information is simply spread by irresponsible people.
